# Transcriptome analysis of thermophilic methylotrophic *Bacillus methanolicus* MGA3 using RNA-sequencing provides detailed insights into its previously uncharted transcriptional landscape

**DOI:** 10.1186/s12864-015-1239-4

**Published:** 2015-02-14

**Authors:** Marta Irla, Armin Neshat, Trygve Brautaset, Christian Rückert, Jörn Kalinowski, Volker F Wendisch

**Affiliations:** Genetics of Prokaryotes, Faculty of Biology & Center for Biotechnology, Bielefeld University, Universitätsstr. 25, 33615 Bielefeld, Germany; Microbial Genomics and Biotechnology, Center for Biotechnology, Bielefeld University, Universitätstr. 27, 33615 Bielefeld, Germany; Department of Molecular Biology, SINTEF Materials and Chemistry, Sem Selands vei 2, 7465 Trondheim, Norway; Department of Biotechnology, Norwegian University of Science and Technology, Sem Sælands vei 6/8, 7491 Trondheim, Norway; Technology Platform Genomics, Center for Biotechnology, Bielefeld University, Universitätsstr. 27, 33615 Bielefeld, Germany

**Keywords:** *Bacillus methanolicus*, Methylotrophy, RNA-sequencing, Transcriptome analysis, Conserved sequence motifs, Operon structures, Regulatory RNA, Transcript abundances, Transcriptional start sites, Ribosome binding sites

## Abstract

**Background:**

*Bacillus methanolicus* MGA3 is a thermophilic, facultative ribulose monophosphate (RuMP) cycle methylotroph. Together with its ability to produce high yields of amino acids, the relevance of this microorganism as a promising candidate for biotechnological applications is evident. The *B. methanolicus* MGA3 genome consists of a 3,337,035 nucleotides (nt) circular chromosome, the 19,174 nt plasmid pBM19 and the 68,999 nt plasmid pBM69. 3,218 protein-coding regions were annotated on the chromosome, 22 on pBM19 and 82 on pBM69. In the present study, the RNA-seq approach was used to comprehensively investigate the transcriptome of *B. methanolicus* MGA3 in order to improve the genome annotation, identify novel transcripts, analyze conserved sequence motifs involved in gene expression and reveal operon structures. For this aim, two different cDNA library preparation methods were applied: one which allows characterization of the whole transcriptome and another which includes enrichment of primary transcript 5′-ends.

**Results:**

Analysis of the primary transcriptome data enabled the detection of 2,167 putative transcription start sites (TSSs) which were categorized into 1,642 TSSs located in the upstream region (5′-UTR) of known protein-coding genes and 525 TSSs of novel antisense, intragenic, or intergenic transcripts. Firstly, 14 wrongly annotated translation start sites (TLSs) were corrected based on primary transcriptome data. Further investigation of the identified 5′-UTRs resulted in the detailed characterization of their length distribution and the detection of 75 hitherto unknown *cis-*regulatory RNA elements. Moreover, the exact TSSs positions were utilized to define conserved sequence motifs for translation start sites, ribosome binding sites and promoters in *B. methanolicus* MGA3. Based on the whole transcriptome data set, novel transcripts, operon structures and mRNA abundances were determined. The analysis of the operon structures revealed that almost half of the genes are transcribed monocistronically (940), whereas 1,164 genes are organized in 381 operons. Several of the genes related to methylotrophy had highly abundant transcripts.

**Conclusion:**

The extensive insights into the transcriptional landscape of *B. methanolicus* MGA3, gained in this study, represent a valuable foundation for further comparative quantitative transcriptome analyses and possibly also for the development of molecular biology tools which at present are very limited for this organism.

**Electronic supplementary material:**

The online version of this article (doi:10.1186/s12864-015-1239-4) contains supplementary material, which is available to authorized users.

## Background

*Bacillus methanolicus* MGA3, isolated from freshwater marsh soil, is an aerobic, Gram-positive, endospore-forming, rod-shaped bacterium auxotrophic for biotin and vitamin B_12_ [1,2]. This thermophilic organism grows in a temperature range between 37 and 60°C with an optimum between 50–53°C [1,2]. *B. methanolicus* MGA3 is able to utilize methanol as its sole carbon and energy source *via* the RuMP assimilation cycle. Moreover, *B. methanolicus* was reported to secrete 55 grams of glutamate per liter in fed-batch cultures with methanol as sole carbon and ammonium as nitrogen source [3,4]. These characteristics indicate that *B. methanolicus* is a valuable candidate for amino acid production from methanol which in turn is an interesting alternative to the conventional feedstocks used in the biotechnological industry, *i.e.* mainly molasses and starch hydrolysates. Methanol is advantageous since it is abundant, its price does neither depend on the climate nor on agricultural politics, it is a pure compound and it reduces the risk of contamination in fermentations due to its toxicity towards numerous microbial species [5,6]. Fermentations with the comparatively reduced methanol are characterized by high oxygen demands and heating of bioreactors causing high cooling costs. However, this is less relevant for the thermophilic *B. methanolicus* [5,6].

Most molecular biology work on *B. methanolicus* has been devoted to enzymes relevant to methylotrophy. *B. methanolicus* possesses three active variants of NAD-dependent methanol dehydrogenase, two chromosome-encoded and one pBM19-encoded, for oxidation of methanol to formaldehyde [7,8], and one activator protein ACT [9]. Formaldehyde is assimilated in the RuMP cycle. The enzymes for condensation of formaldehyde with ribulose to fructose 6-phosphate, 3-hexulose 6-phosphate synthase and 6-phospho-hexuloisomerase, are only encoded on the chromosome [10]. The majority of genes encoding the enzymes of the RuMP cycle in *B. methanolicus* MGA3 is present in the genome in two copies, chromosome-encoded and pBM19-encoded [10,11]. Although all of the genes coding for the transketolase variant of the regeneration phase have their chromosomal version, pBM19-cured *B. methanolicus* MGA3 strain MGA3-A6 shows a methylotrophy deficient phenotype [10]. The five RuMP cycle genes and *mdh* encoded on pBM19 are induced during growth on methanol. The variants of the enzymes do not always differ in their biochemical characteristics, *e.g.* the transketolases [12]. By contrast, the fructose 1,6-bisphosphatases differ since the major fructose 1,6-bisphosphatase GlpX^C^ is encoded on the chromosome, while pBM19-encoded GlpX^P^ is also active as sedoheptulose 1,7-bisphosphatase in the eponymous regeneration variant of the RuMP cycle [13]. Similarly, whereas both fructose 1,6-bisphosphate aldolases FBA^C^ and FBA^P^ are also active as sedoheptulose 1,7-bisphosphate aldolases, their kinetic parameters allowed to distinguish FBA^C^ as major glycolytic FBA and FBA^P^ as major gluconeogenic FBA [14].

Sequencing of the *B. methanolicus* MGA3 genome, comparative DNA microarray transcriptome analyses and proteome studies have increased our understanding of the genetic and regulatory background for methanol metabolism in *B. methanolicus* MGA3 [10,11,15,16]. However, to date a comprehensive transcriptome analysis of *B. methanolicus* MGA3 is still missing. Therefore, transcriptome sequencing (RNA-seq) [17,18] was here used for the complete analysis of the mostly uncharted transcriptional landscape of *B. methanolicus* MGA3. Cultivations of *B. methanolicus* MGA3 were performed in several different conditions in order to capture a wide range of transcripts. RNA-seq data for the analysis of the primary transcriptome and the whole transcriptome were generated to gain insights about the exact positions of transcription start sites, length distribution of 5′-UTRs, consensus promoter and ribosome binding site (RBS) motifs, transcriptional organization of operons, and mRNA abundances. Moreover, *cis-*regulatory RNA elements and novel transcripts in intergenic regions, intragenic sense transcripts of annotated genes as well as antisense transcripts located within known genes were identified.

## Methods

### Cultivation of *B. methanolicus* MGA3 in different growth media

*B. methanolicus* wild-type strain MGA3 was used as the model organism in all growth experiments. The fermenter minimal medium was a derivative of MVcMY medium but instead of vitamin solution, 6 mg/liter d-biotin and 0.01 mg/liter vitamin B_12_ were used [19]. If not stated differently 200 mM methanol was used as carbon source both for flask and fermenter cultivations. The overnight cultures grown in 500 ml shake flasks with 50 ml of working volume of MVcMY minimal medium at 50°C and 200 rpm as described by Brautaset and coworkers [4] were used as inoculum for fermentations at a start optical density (OD_600_) of 0.15. The fermentations were performed in 1 L Biostat Q bioreactors with working volume of the minimal medium of 800 ml (Sartorius, Göttingen, Germany). The standard cultivation conditions were: temperature at 50°C, the dissolved oxygen level at 30% and the pH maintained by automatic addition of 12.5% NH_4_OH (wt/vol) at 6.5. An antifoam agent (Antifoam 204, Sigma-Aldrich, St. Louis, MO, USA) was added at an initial concentration of 0.1% (vol/vol). Fermentations were performed with initial agitation at level of 120 rpm and with no initial aeration. The level of dissolved oxygen was controlled by automatic adjustment of the aeration rate up to 0.75 NL∙min^−1^ and followed by an increase of the stirrer speed up to 1200 rpm.

In order to induce the transcription of as many genes as possible, *B. methanolicus* was cultivated in 16 different conditions; 13 cultivations in bioreactors and 3 cultivations in shake flasks. For induction of pH shock response the bacterium was cultivated in a pH range between 5.5 and 7.5 with 0.5 intervals, for oxygen stress conditions the dissolved oxygen was maintained at levels 5%, 20% and 50%, and osmotic stress was induced by addition of 300 mM sorbitol or 160 mM NaCl. Additionally, 50 mM mannitol or 50 mM glucose were used as carbon sources and 16 mM glutamine was used as sole nitrogen source. For the latter fermentation, 1 N NaOH was used to maintain pH at a level of 6.5. Apart from above described changes, the fermentations were performed in standard conditions. Additionally, three flask cultivations were performed in minimal medium MVcMY with 200 mM methanol, 50 mM glucose or 50 mM mannitol as the sole carbon source. In each case, 20 mL of sample was collected in the exponential phase to 50 mL falcons filled with ice and centrifuged at 4°C for 10 min at 3.220 x g. For the standard cultivation conditions the samples were additionally collected at the beginning and at the end of the exponential phase. The resulting cell pellets were frozen in liquid nitrogen and stored at −80°C until further use.

### Total RNA isolation

For the extraction of total RNA the NucleoSpin RNA isolation kit (MACHEREY-NAGEL, Düren, Germany) and the RNase-free DNase set (QIAGEN, Hilden, Germany) were used according to the manufacturer’s instructions. The total RNA was isolated individually for each cultivation condition. The quality and quantity of the isolated RNA was assessed by PCR with gene-specific primers (Additional file 1: Table S1) and with capillary gel electrophoresis (Agilent Bioanalyzer 2100 system using the Agilent RNA 6000 Pico kit; Agilent Technologies, Böblingen, Germany). The extracted RNA samples were pooled in equal parts and the pool of total RNA was subsequently used for the preparation of two different cDNA libraries.

### Preparation of two different cDNA libraries for high-throughput sequencing

The cDNA libraries of *B. methanolicus* MGA3 were prepared according to two different protocols. One approach focused on the enrichment of 5′-ends of primary transcripts, while the other method allowed the analysis of the whole transcriptome. Both cDNA library preparation protocols have been previously described in detail [17]. In the present study, the experimental workflow was changed at two steps. The first difference is that the amplification of cDNA fragments was customized by adding the PCR additive betaine to a final concentration of 2.7 M in each PCR reaction. Secondly, after cDNA amplification the two libraries were purified and size-selected *via* gel electrophoresis for fragment sizes between 100 and 800 bp. The cutoff of 100 bp was chosen to reduce adapter dimers in the finished library. Due to the fact that the preparation workflow involves the use of two adapters, which together have a length of 66 nt, only transcripts smaller than 40 nt are not present in the final RNA seq data. Subsequently, each cDNA library was sequenced according to the manufacturer’s instruction on a single flow cell using TruSeq kits (Illumina, San Diego, CA, USA) on a MiSeq Desktop Sequencer system (Illumina, San Diego, CA, USA). Sequencing was performed in single-read mode with 50 nt read length for the enriched 5′-ends cDNA library and in paired-end mode with 25 nt read length for the whole transcriptome cDNA library. The base calling was realized with the Illumina instrument software.

### Mapping of the generated RNA-seq data to reference sequences

In order to perform further analyses on the RNA-seq data, the generated reads were mapped to the *B. methanolicus* MGA3 reference sequences of the chromosome and the two plasmids pBM69 and pBM19 (GenBank accession no. CP007739, CP007740 and CP007741). For this, two different strategies were utilized for the enriched 5′-ends of primary transcripts RNA-seq data and the whole transcriptome data set. In a preprocessing step, the last bases of all sequence reads from the enriched 5′-ends approach were trimmed to a final length of 20 nt. This was mainly done because only the read starts of this data set are important for subsequent analyses, but also because of the higher error rate which is often displayed by the last bases of sequence reads [20]. Next, these trimmed reads were mapped to the *B. methanolicus* MGA3 reference sequences using the exact mapping algorithm SARUMAN [21] and allowing a single mismatch to occur in the alignment of each read. In case of the reads belonging to the whole transcriptome RNA-seq data set, the forward and reverse read were combined to one read, containing the reference sequence as an insert, if both reads were present and in inverse orientation in a maximal distance of 1 kb to each other. Sequence reads not fulfilling this criterion were retained as single mapping reads. Paired mappings with a distance larger than 1 kb were discarded from subsequent analyses. For the visualization of the mapped short reads, the Java software ReadXplorer [22] was used.

### Determination of putative transcription start sites using 5′-ends of enriched primary transcripts

**Figure 1:**
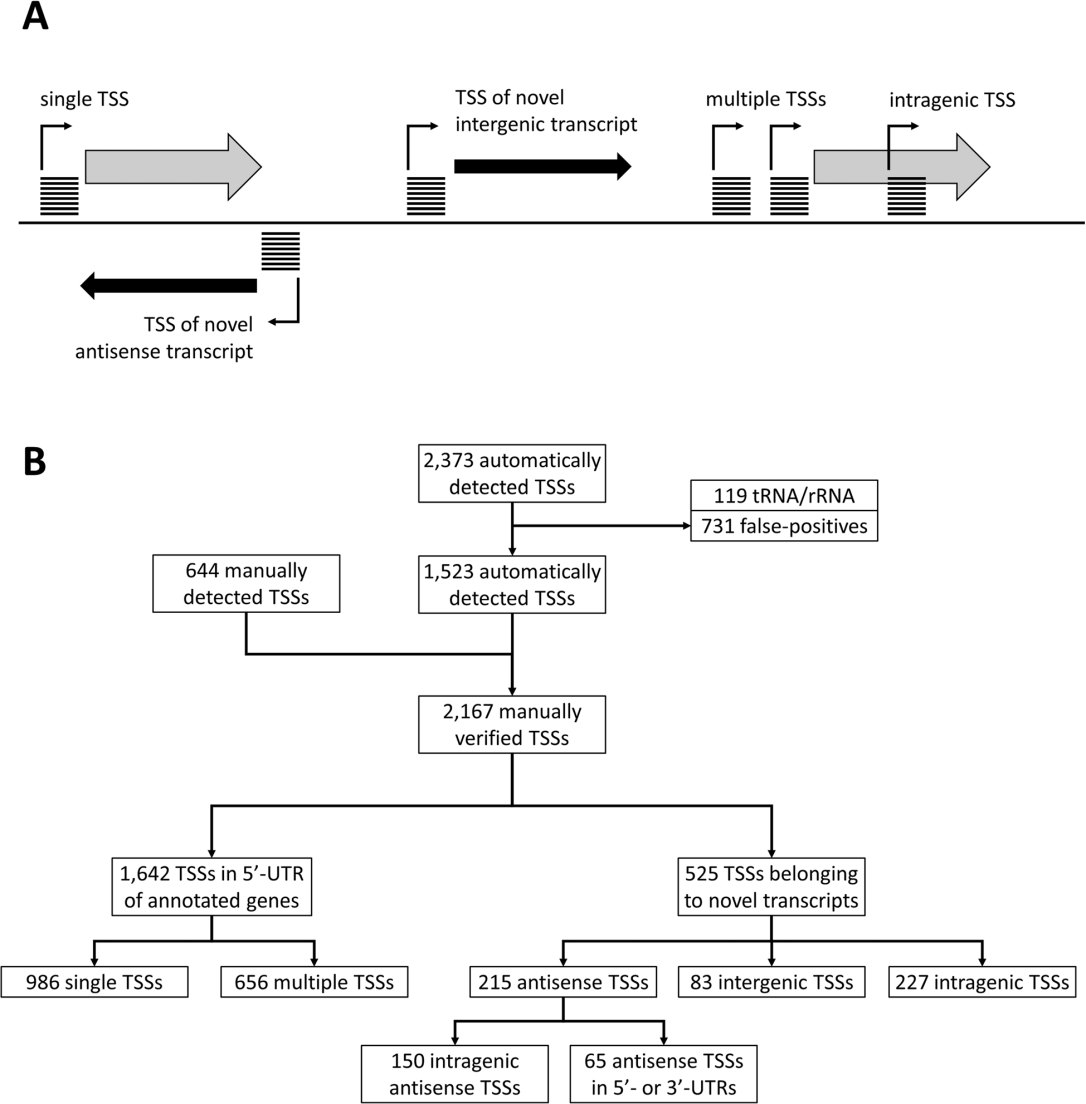
**Overview of the classification of putative TSSs within the** ***B. methanolicus*** **MGA3 genome sequence. (A)** Schematic illustration of the different categories which were used for the classification of TSSs based on the respective genomic context. Putative TSSs are depicted as angled black arrows and are identified as described in section “Preparation of two different cDNA libraries for high-throughput sequencing” using the read starts obtained from RNA-seq data of enriched 5′-ends cDNA library. TSSs located in the upstream region and in coding direction of known CDSs (gray arrows) were classified as single TSSs or multiple TSSs. All TSSs overlapping in sense direction with known CDSs were categorized as novel intragenic TSSs. TSSs without annotated features downstream were classified as novel intergenic TSSs (black arrow), while TSSs antisense to annotated CDSs were classified as novel antisense TSSs (black arrow). **(B)** Process of TSSs analysis which includes the identification, filtering, manual verification and classification of putative TSSs. After manual inspection TSSs that belong to rRNA/tRNA and false-positive TSSs were removed from the automatically detected set, whereas the manually detected TSSs were added. The complete set of verified TSSs was divided into subsets depending on their genomic context.

The identification of putative transcription start sites was performed based on the method described previously [17], but with a recently developed transcriptome analysis module which will be integrated into the software ReadXplorer in the near future [www.readxplorer.org, 22]. To determine putative TSSs, the RNA-seq data of enriched native 5′-ends was used. First, for each strand and position of the *B. methanolicus* MGA3 reference sequences, all reads starts at each genomic position were counted. Putative TSSs were automatically and systematically called if the read starts at the analyzed genomic position fulfilled three criteria. A putative TSS was identified if the number of read starts at the genomic position exceeded the background threshold *T* and if the ratio of read starts at this position to read starts at the previous position was higher than the ratio *R*. Additionally, the putative TSS was only automatically identified if it was located within a maximal distance *X* between the putative TSS and the next TLS. For every identified candidate TSS, additional information on the genomic vicinity regarding next upstream, downstream, and overlapping features on both strands were taken into account. In particular, the gene identifier, gene type (protein-coding gene, rRNA, tRNA), TSS distance to feature start, and TSS distance to feature stop were reported. Additionally, the putative TSSs set, which was obtained by automated analysis, was manually cross-checked with the complete enriched 5′-ends dataset. This revision allowed to disregard false-positive TSSs and to add putative true-positives which are below the automatic detection limit. A putative TSS was assumed to be false-positive and disregarded if no clear accumulation of read starts was observed at the particular genomic position and additionally the putative TSS was detected within an uneven gradient of accumulated read starts. The described combination often applied to putative TSSs detected within a coding region and/or with a relatively high amount of accumulated reads (>100), where the parameters used for automated TSSs detection are not effective. A putative TSS was manually added if only a single read difference to one of the thresholds *T* or *R* was observed and if additionally the whole transcriptome RNA-seq data confirmed the assumption of a valid transcript at the examined genomic position. In addition, most novel intergenic features were manually added as these understandably did not match the chosen maximal distance cutoff between TSSs and next TLSs. Following the manual verification, the determined set of putative TSSs was divided into subsets depending on their genomic context within the annotated genome of *B. methanolicus* MGA3 (Figure 1).

### Identification of operon structures using whole transcriptome cDNA library RNA-seq data

To determine operon structures of *B. methanolicus* MGA3, the generated whole transcriptome cDNA library RNA-seq data was used. In the present study, an approach was applied which is based on a previously described method [17] and which soon will be integrated into the software ReadXplorer [www.readxplorer.org, 22]. For the identification of operons, it was examined if a gene and the next downstream gene in sense direction are connected by a number of paired mappings exceeding the background threshold *T* for this data set. The background threshold *T* was set to 4 after manual analysis of different values. Thus, two genes oriented in sense direction were combined to an operon unit if at least 4 paired mappings were bridging over the intergenic region of those genes. This process was progressively performed for all genes located on the chromosome and the two plasmids of *B. methanolicus* MGA3. All consecutive gene pairs were combined, whereas the first gene of the operon was always considered to constitute the primary operon. In most cases a TSS could be determined for the first gene of these primary operons. Suboperons were called if TSSs were detected for genes within the primary operon and defined as subset of genes preceded by the TSS present within the primary operon. Additionally, the automatically generated operon set was manually cross-checked with the complete whole transcriptome RNA-seq data. In this context, genes were manually added to an operon if only a single read difference to the thresholds *T* was observed and if additionally the genes were functionally related.

### Detection of conserved sequence motifs in *Bacillus methanolicus* MGA3

To identify conserved sequence motifs for translation starts, ribosome binding sites and promoter regions of *B. methanolicus* MGA3, the motif-finding program Improbizer [23] and the visualization tool WebLogo [24] were used with default settings. The input differed according to the respective analysis context: For the determination of TLSs and promoter motifs all genes with an identified 5′-UTR were considered, while for the RBSs motifs only 5′-UTRs longer than 9 nucleotides were analyzed. The identified motifs are represented in the text by upper or lower case bases dependent on their conservation. An upper case letter is used if the nucleotide occurs in more than 80% of all analyzed sequences, whereas a lower case letter is used, if the base appears in more than 40%, but less than 80% of all cases. If a base occurs less than 40% at a certain position, a lower case n is used.

### Calculation of transcript abundances in *Bacillus methanolicus* MGA3

To determine and compare transcript abundances across different features in *B. methanolicus* MGA3, the generated whole transcriptome data had to be normalized. One of the most basic methods for the normalization of RNA-seq data is referred to as reads per kilobase of coding DNA sequence (CDS) per million mapped reads (RPKM) [25]. To account for fragmentation and PCR biases during the cDNA library preparation, log-RPKM values were determined and used for comparison of transcript abundances in the present study. For this the following calculations were performed:

$$ \overline{x}=\frac{{\displaystyle {\sum}_{p_{i=1}}^N ln\left(r{s}_{p_i}\right)}}{N} $$

For each single nucleotide position *p*_*i*_ belonging to a transcript, the natural logarithm of the number of mapped read starts at that position ($r{s}_{p_i}$) was calculated and added up, if at least one read was mapped. Next, the arithmetic mean $\overline{x}$ of these data was calculated and this number was used in the following formula:

$$ {R}_{norm} = {e}^{\overline{x}}N $$

to finally obtain a normalized quantity of reads per CDS (*R*_*norm*_). This number of normalized reads per CDS was than utilized in the common formula for RPKM determination.

## Results

### Cultivations of *B. methanolicus* MGA3 under different conditions for analysis of the primary and whole transcriptome

The comprehensive characterization of ‘static’ bacterial transcriptomes requires the expression of as many genes as possible in order to obtain a pool of different transcripts. In this study, *B. methanolicus* MGA3 was subjected to 13 bioreactor fermentations and three flask cultivations with different carbon sources (200 mM methanol, 50 mM mannitol and 50 mM glucose) and under various growth conditions. The standard 1-L bioreactor cultivation was carried out at pH 6.5, pO_2_ 30%, temperature 50°C and with 200 mM methanol as carbon source and a working volume of 800 mL. The changes in these standard settings included pH varying between 5.5 and 7.5, pO_2_ at levels of 5%, 20% and 50%, use of 50 mM glucose or mannitol instead of methanol as sole carbon source and 16 mM glutamine as sole nitrogen source, and application of osmotic stress by addition of 300 mM sorbitol or 160 mM NaCl. The samples were taken at three time points (3, 4 and 8 hours with the respective OD_600_ of 1.6, 2.4 and 12.1) during bioreactor cultivation in standard conditions and only at one time point in the exponential phase for the other cultivations (OD_600_ of 2.5 to 6). The growth pattern under the standard conditions (high growth rate of 0.52 h^-1^, decline of biomass concentration after reaching maximum OD_600_ of 13.5 corresponding to 2.97 g CDW/L) was comparable to the one previously described for *B. methanolicus* [26] (Additional file 2: Table S2). During cultivation on glucose as the sole carbon and energy source, sporulation was observed which is in accordance with previous findings [26]. As expected, growth rates were lower under stress conditions (pH, osmotic and oxygen stress) than for standard cultivation conditions. Taken together, these results indicate that *B. methanolicus* MGA3 was submitted to various different growth conditions, thus, quite different gene expression patterns involving most of the genes on the *B. methanolicus* genome may be expected.

### Sequencing of cDNA libraries and mapping to the *B. methanolicus* MGA3 reference sequences

After RNA isolation and pooling, two cDNA libraries (whole transcriptome and enriched for 5′-ends of primary transcripts) were prepared as described previously [17]. The cDNA libraries were sequenced on a single flow cell on a MiSeq Desktop Sequencer system. About 3.28 million sequence reads were obtained from enriched 5′-ends of primary transcripts and about 4.24 million sequence reads for the whole transcriptome cDNA library (Table 1). Subsequently, the obtained reads were mapped to the chromosome and the two plasmids of *B. methanolicus* MGA3 using the exact alignment algorithm SARUMAN [21]. About 1.30 million of 3.28 million sequence reads for the enriched 5′-ends of primary transcripts library and about 4.20 out of the 4.24 million sequence reads of the whole transcriptome library were mapped to the reference sequences. Of those mapped reads, 1.25 million reads from enriched 5′-ends of primary transcripts and 2.60 million reads from the whole transcriptome mapped uniquely. Reads of the whole transcriptome data were combined to one read with the reference sequence as insert, if the forward and reverse read were present and maximal distance to each other was 1 kb (Table 1).

**Table 1:** **Sequencing and mapping results for the cDNA libraries of** ***B. methanolicus*** **MGA3**

	**Reference**	**5′-ends of primary transcripts**	**Whole transcriptome**
Sequence reads		3,278,605	4,241,887
Mapping reads	Chromosome	1,189,365	3,857,244
	pBM19	91,185	315,572
	pBM69	17,709	28,341
	**Total**	**1,298,259**	**4,201,157**
Unique matches (single reads)	Chromosome	1,141,761	942,678
	pBM19	91,169	68,844
	pBM69	17,686	7,793
	**Total**	**1,250,616**	**1,019,315**
Unique matches (combined reads)	Chromosome	-	1,444,584
	pBM19	-	123,354
	pBM69	-	10,172
	**Total**	-	**1,578,110**

### Detection of putative transcription start sites from RNA-seq data of enriched 5′-ends of primary transcripts

Putative TSSs were derived from the mapped read starts of enriched 5′-ends of primary transcripts. Based on background threshold *T* (empirically set to 6), ratio *R* (6) and distance cutoff *X* of 500 bp that showed a good signal-to-noise ratio and specificity after manual inspection of randomly selected TSSs from the result set, 2,373 TSSs were detected (Figure 1). After a complete review of the TSSs set and comparison to the raw RNA-seq data set, 644 manually determined TSSs were added to the group of TSSs, while 731 false-positive TSSs and 119 TSSs located upstream of tRNA or rRNA genes were removed. These 2,167 manually verified TSSs were assigned to subgroups of TSSs that are located in the 5′-UTR of annotated genes (1,642) and TSSs that belong to novel transcripts (525). The 1,642 TSSs found in the 5′-UTRs of annotated genes were further categorized into transcripts for which only one or several TSSs could be determined, thus resulting in a set of 986 single TSSs and a set of 656 multiple TSSs, originating from 283 genes (Figure 1). Of the 525 TSSs belonging to novel transcripts, 215 were located in antisense orientation to an annotated gene or its untranslated region, 83 TSSs were intergenic and 227 were intragenic TSSs. The intragenic TSS within the first half of CDSs were used to correct 14 CDSs starts, which were previously wrongly annotated (Additional file 3: Table S3).

### Determination and length distribution of 5′-untranslated regions in *B. methanolicus* MGA3

**Figure 2:**
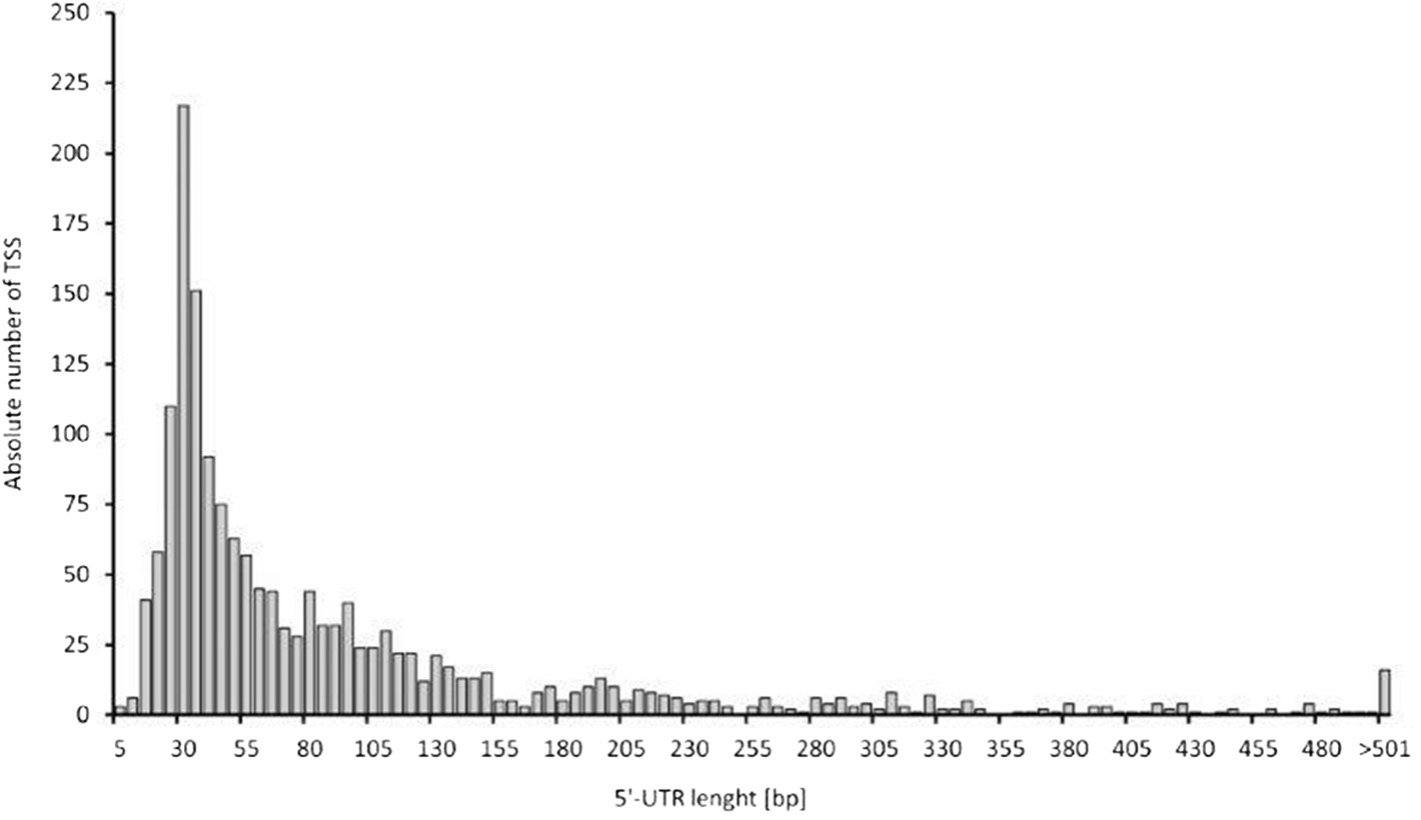
**Absolute number of identified transcription start sites in correlation to the length of their 5′-untranslated regions (5′-UTRs).** The 1,642 TSSs located upstream or in coding direction of known CDSs were used to determine the length of the 5′-UTRs for each CDS. The 5′-UTR length was calculated as the distance between an identified TSS to the next TLS. The absolute number of TSSs is grouped in 5 bp intervals of 5′-UTR lengths (1–5, 6–10, 11–15 etc.), whereas the most distant right bar represents all 5′-UTRs longer than 500 bases.

The distances of 1,642 TSSs belonging to the leader regions of protein-coding genes to the next TLSs revealed a median 5′-UTR length of 51 nt (Figure 2). More than 99% of the transcripts analyzed in this study have 5′-UTR leader sequences of ≥ 10 nt. 22% of transcripts showed a 5′-UTR length of 26–35 nt and 25% of transcripts contain leader sequences longer than 100 nt, which suggests that they might contain regulatory RNA structures [27]. Only six transcripts (<0.5%) possess a 5′-UTR shorter than 10 nt, which implies that they do not retain a (complete) RBS. However, no leaderless transcript was found for *B. methanolicus* MGA3 since all transcripts had 5′-UTRs ≥ 2 nt. Only two transcripts showed leaders shorter than 5 nt. The corresponding genes are *BMMGA3_05465* encoding the CtaG protein which participates in the formation of active cytochrome caa3 and *sigF* encoding the sporulation-specific sigma factor *σ*^F^ [28].

### Identification of ribosome binding sites in *B. methanolicus* MGA3

**Figure 3:**
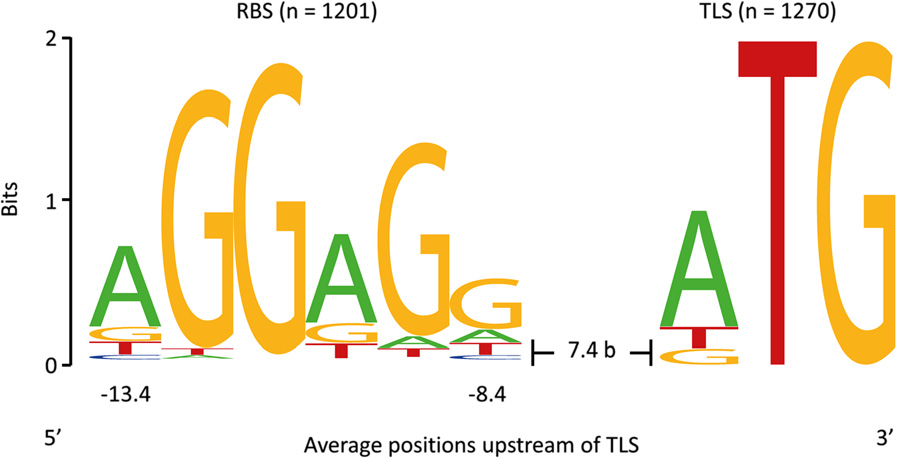
**Distribution of nucleotides within the ribosome binding sites and translation starts of** ***B. methanolicus*** **MGA3.** The analysis of the nucleotide distribution in translation start sites and ribosome binding sites of *B. methanolicus* MGA3 was based on the TLS and upstream regions of genes for which a 5′-UTR was identified in the present study. The conserved TLSs and RBSs motifs were determined by using the motif-finding program Improbizer [23]. The conservation of a specific nucleotide at certain position is measured in bits and represented in the illustration by the size of the nucleotide. The depicted sequence logo was created with the software WebLogo [24].

For the determination of the nucleotide distribution in TLSs of *B. methanolicus* MGA3, the initiation codons of 1,270 genes, for which a 5′-UTR was identified (section “Detection of putative transcription start sites from RNA-seq data of enriched 5′ ends of primary transcripts”), were analyzed. For genes with more than one TSS, the upstream sequence of the respective gene was only included once in the analysis. From this set, 1,264 upstream regions of genes with a 5′-UTR longer than 9 nt were used to identify a conserved ribosome binding site sequence using the tools Improbizer [23] and WebLogo [24]. The determined RBS motif of *B. methanolicus* MGA3 (Figure 3) was present in 1,201 of 1,264 upstream regions (95.0%). The preferred translational start codon was ATG (959 genes, 75.5%), followed by TTG (177 genes, 13.9%) and GTG (134 genes, 10.6%), while CTG was not found in such position. The determined RBS motif in *B. methanolicus* MGA3 is aGGaGg with the three guanines depicted in capital letters being present in approximately 90% of the analyzed sequences (Figure 3). The distance between the RBSs and the TLSs varies between 5 to 10 nucleotides (97.1%) with an average spacing of 7.4 bases. The assumed optimal interval of 7 or 8 bases accounts for 58.5% of the determined spacers in *B. methanolicus* MGA3.

### Identification of promoter motifs in *B. methanolicus* MGA3

**Figure 4:**
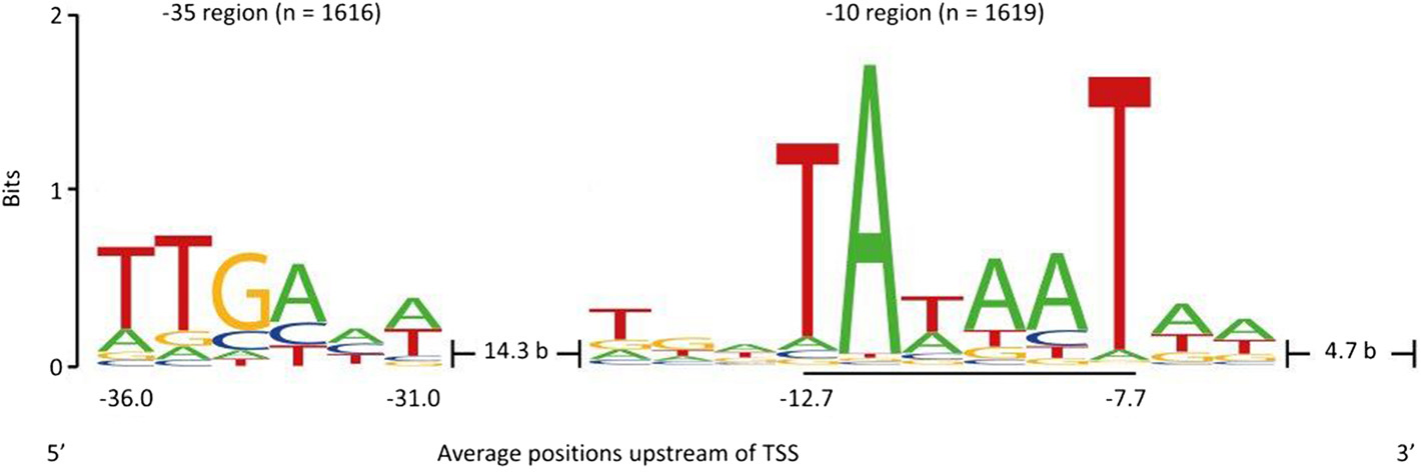
**Distribution of nucleotides within the −10 and −35 regions of** ***B. methanolicus*** **MGA3 promoter regions.** The conserved sequences were determined by using the Improbizer motif-finding program [23]. For this analysis, the upstream regions of the 1,642 TSSs located in the 5′-UTR of annotated protein-coding genes were used. Conserved -10 motifs were detected in 1,619 sequences (98.6%), whereas 1,616 of the analyzed sequences contributed to identification of the -35 motif (98.4%). The conservation of a specific nucleotide at certain position is measured in bits and represented in the illustration by the size of the nucleotide. The hexamer of the core -10 region is underlined. The position values below the nucleotides are represented in relation to the positions of the identified TSSs, while the two spacers represent the mean distance between extended -10 region and TSS or -10 and -35 region, respectively. The depicted sequence logo was created with the software WebLogo [24].

The upstream regions of the 1,642 TSSs, which were identified at the 5′-UTR of annotated genes (see section “Detection of putative transcription start sites from RNA-seq data of enriched 5′ ends of primary transcripts”) were searched for conserved motifs within 70 bases upstream of each TSS using Improbizer [23]. The −10 hexamer sequence TAtaaT was identified in 1,619 of the upstream sequences (98.6%) (Figure 4) and in 33% of the sequences the motif TGN was present. The distance between an identified -10 region to the corresponding TSS ranges from 4 to 10 nt and is in average 6.7 bases in length. Upstream of an identified −10 hexamer sequence, a weakly conserved −35 motif ttgana was found in 1,616 (98.4%) upstream sequences (Figure 4). The first three bases of the motif are present in approximately 70% of the sequences and the following three bases in about 46%. The average distances between the −10 and the -35 regions was 16.6 bases in *B. methanolicus* MGA3.

When the genes with 1,642 detected upstream regions were subdivided into the four COG (Clusters of Orthologous Groups) categories: information storage and processing (1), cellular processes and signaling (2), metabolism (3) and unknown function or poorly characterized (4), nearly the same −10 and −35 motifs as described above were present in each group (data not shown). Slight deviations were detected only for the weaker conserved positions of the two motifs. In group 1, a weakly conserved guanine was observed at the second position upstream of the −10 region. In groups 1 and 2, thymine at the last position of the -35 region is slightly more conserved than adenine. The first three bases (ttg) of the −35 region were marginally less conserved in group 4.

The 21 transcripts with the highest abundance (section “Transcript abundances of *B. methanolicus* MGA3”) and with an available upstream region showed conserved −10 and -35 elements, however, certain differences to the above described consensus motifs were found: The third position of the −10 hexamer showed no conservation at all (TAnaaT), while the first three bases of the −35 region (tTg) and especially the second position were slightly more conserved (data not shown).

### Determination of operon structures based on the whole transcriptome data set

**Figure 5:**
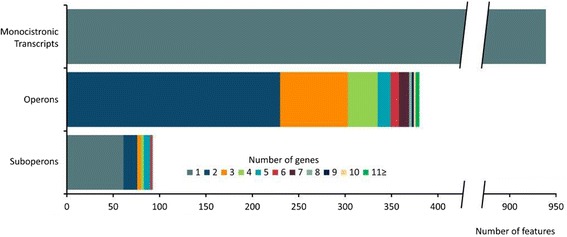
**Analysis of operon structures and comparison of the number of genes assigned to monocistronic transcripts, primary operons and suboperons, identified in** ***B. methanolicus*** **MGA3.** The bars represent the different categories of transcripts. Within each category the number of genes is highlighted with a color code as depicted in the legend below.

Using the paired-end sequencing reads from the whole transcriptome approach, an automated search for operon structures in *B. methanolicus* MGA3 was performed. The neighboring genes organized on the chromosome in sense direction were considered to compose a primary operon if they were connected by at least 4 combined read pairs. If TSSs were found within primary operon structures, suboperons were indicated. Upon manual analysis of the RNA-seq data and by literature search, in 28 cases single genes were added to automatically detected operons. In total, 940 monocistronic transcripts were found in the transcriptome of *B. methanolicus* MGA3 and 1,164 genes were components of 381 operons with 94 suboperons (Figure 5). Thus, transcripts were detected for 2,092 of 3,330 (62.8%) annotated genes in the genome of *B. methanolicus* MGA3. Most operons (60.5%) consist of two genes and 12 operons comprised 8 or more genes (Table 2). The two largest operons each comprise 31 genes. The *flgB* operon is composed of 31 genes responsible for cell movement and chemotaxis in identical gene arrangement as found in *B. subtilis* [29]*.* The *rpsJ* operon with 26 genes coding for ribosomal proteins and five genes of various functions is also present in identical transcriptional gene organization in *B. subtilis* [30]. Only one of the 12 large operons, possibly involved in biosynthesis of amino sugars and their subsequent polymerization, does not have an equivalent in the genome of *B. subtilis*, but these genes can be found in *Bacillus firmus*, *Anoxybacillus tepidamans* and some *Geobacillus* species.

**Table 2:** **Largest identified primary operons on the** ***B. methanolicus*** **MGA3 chromosome**

**Genes**	**No. of genes**	**Strand**	**Putative gene functions**	**Transcriptional organization in** ***B. subtilis*** ^**1**^
*BMMGA3_00690*-*BMMGA3_00840*	31	+	Various, mainly translation and ribosomal structure	Similar, [31]
*BMMGA3_06100*-*BMMGA3_06250*	31	+	Movement and chemotaxis	Similar, [29]
*BMMGA3_01635*-*BMMGA3_01690*	12	+	Purine biosynthesis	Similar, [32]
*BMMGA3_15075*-*BMMGA3_15130*	12	-	Amino sugars biosynthesis and polymerization	No similar operon present
*BMMGA3_05780*-*BMMGA3_05825*	10	+	Pyrimidine biosynthesis	Similar, [33]
*BMMGA3_15975*-*BMMGA3_16020*	10	-	ATP synthesis	[8/10] + 1, [34]
*BMMGA3_12730- BMMGA3_12770*	9	-	Biosynthesis of branched amino acids	[8/9] + 1, [35]
*BMMGA3_01305*-*BMMGA3_01345*	9	+	Control of SigB activity, general stress response	[7/9] + 1, [36]
*BMMGA3_02555*-*BMMGA3_02595*	9	+	Sulfate reduction and activation, siroheme biosynthesis	[8/9]* + 2, [37]
*BMMGA3_00415*-*BMMGA3_00450*	8	+	Folic acid biosynthesis	[7/9]^§^ [38]
*BMMGA3_05870*-*BMMGA3_05905*	8	+	Various, mainly translation, ribosomal structure, signal transduction	[3 + 3/7]^‡^ +2, [39,40]
*BMMGA3_14725*-*BMMGA3_14760*	8	-	Histidine biosynthesis	Similar, [41]

^1^ Genes in the same transcriptional organization as *B. methanolicus* MGA3 are depicted in square brackets. The number of transcripts that are present in the *B. subtilis* genome, but are not associated to the respective operon are indicated after the plus sign.

* In comparison to the *B. methanolicus* MGA3 the gene order is altered.

^§^ In *B. subtilis*, the operon contains one additional gene.

^‡^ In *B. subtilis*, the genes are organized in two separate operons with.

### Identification of novel transcripts in *B. methanolicus* MGA3

Altogether, 525 TSSs belonging to novel transcripts (section “Detection of putative transcription start sites from RNA-seq data of enriched 5′ ends of primary transcripts”) were detected by analysis of the primary transcriptome data set and classified into three categories according to their genomic context: intergenic (83), intragenic (227) or antisense (215). In addition, the whole transcriptome RNA-seq data were used to validate the novel transcripts and to determine their respective 3′-ends. When sequences downstream of the 83 intergenic TSSs were analyzed using ORF finder, BLAST and the Rfam database [42], 27 novel intergenic genes were identified, 24 of which coded for small RNAs (sRNA) of known or unknown function. Three novel genes encoding small and so far not annotated proteins in the *B. methanolicus* MGA3 genome were also found (Table 3 and Additional file 4: Table S4). Furthermore, 11 gene remnants with similarities to at least one homologous protein in another species were found (data not shown). Among the newly identified small RNAs was the small cytoplasmic RNA scRNA (named 4.5S in *E. coli*). The scRNA identified in *B. methanolicus* MGA3 is approximately 337 nucleotides in length and is a member of the evolutionarily conserved signal-recognition-particle-like RNA family [43]. Moreover, RNase P RNA (425 nt), two copies of the 6S RNA, tmRNA, which is part of the bacterial ribonucleoprotein complex, and the SR4 RNA, which belongs to a novel toxin-antitoxin system [44], were identified.

**Table 3:** **Novel transcripts with known function identified in** ***B. methanolicus*** **MGA3**

**Name**	**Locus tag**	**Class**	**Feature start**	**Feature stop**	**Length**	**Strand**
4.5S RNA/scRNA	*BMMGA3_s0190*	sRNA	27,291	27,628	307	+
RNase P RNA	*BMMGA3_s0200*	sRNA	2,087,761	2,087,336	425	-
6S RNA/SsrS	*BMMGA3_s0210*	sRNA	2,278,029	2,277,818	211	-
6S RNA/SsrS	*BMMGA3_s0220*	sRNA	2,437,070	2,436,840	230	-
tmRNA/SsrA	*BMMGA3_s0230*	sRNA	2,890,631	2,890,214	417	-
SR4 RNA	*BMMGA3_s0240*	sRNA	1,925,025	1,924,851*	174	-
*bsrG*	*BMMGA3_9642*	Protein	1,924,777	1,924,908	132	+

*The stop of this feature was approximated based on the published sequence in *B. subtilis* [44] and the terminator predication with ARNold [45].

For 225 of the 227 intragenic TSSs an intragenic sense transcript was found (Additional file 5: Table S5). Interestingly, about one third of the intragenic TSSs were found within operons. Their promoter motifs were found to be nearly identical to the -35 and -10 elements recognized by the housekeeping sigma factor (data no shown).

For 152 of the 215 antisense TSSs transcripts longer than 20 nt were found (Additional file 6: Table S6). Of those, 114 (75.0%) were *cis-*antisense RNAs that overlap with coding regions on the complementary strand, 19 (12.5%) were located antisense to a 5′- or 3′-UTR of an annotated gene and 19 (12.5%) were located antisense to a coding region and additionally overlapped the respective 5′- or 3′-UTR. The 152 antisense genes are preceded by a conserved housekeeping sigma factor −10 motif, but lack conserved -35 motifs (data no shown). The majority of them are genes of unknown function or only poorly characterized (47%) or involved in metabolism (24%). Interestingly, besides these 152 antisense genes, the genome of *B. methanolicus* contains 11 transcribed 5′-UTRs located antisense to the 5′-UTR or the coding region of a neighboring gene.

### Uncovering of *cis*-regulatory RNA elements by the analysis of 5′-UTRs

Manual inspection of 342 identified 5′-UTRs longer than 100 nucleotides (the longest leader sequence was chosen if multiple TSSs were found) and comparison to the Rfam database [42] revealed 75 putative *cis-*regulatory elements present in 68 different 5′-UTRs (Tables 4, 5, 6 and 7). Remarkably, two consecutive *cis-*regulatory elements were present in seven 5′-UTRs. 41 putative riboswitches were found (Table 4) including those likely recognizing adenosylcobalamin (AdoCbl), flavin mononucleotide (FMN), S-adenosylmethionine (SAM), thiamine pyrophosphate (TPP), glycine, lysine, purines, 7-aminoethyl 7-deazaguanine (preQ1), cyclic diguanylate monophosphate (cyclic di-GMP), cyclic diadenylate monophosphate (c-di-AMP), and glucosamine-6-phosphate (GlcN6P). Three putative cobalamin riboswitches were upstream of operons for cobalamin transport and biosynthesis, ribonucleotide-diphosphate reductase NrdAB and a B_12_-independent methionine synthase (Table 4). Putative FMN riboswitches are located in front of the *ribD* operon for riboflavin biosynthesis and the *fmnP* gene encoding a riboflavin transporter. The *tenI* and *thiD* operons, and *thiW* thiamine transporter gene are preceded by putative TPP riboswitches. Moreover, putative TPP riboswitches are located upstream of two similar versions of an operon containing taurine ABC transporter genes *tauB* and *tauC*. Putative glycine riboswitches are located upstream of the glycine utilization operon *gcvT* and the gene *BMMGA3_03000* encoding an uncharacterized protein. A lysine riboswitch precedes the putative arginine/ornithine antiporter gene (Table 4). In *B. methanolicus* MGA3*,* 4 purine riboswitches were found upstream of the *pur* operon for *de novo* biosynthesis of purines, the *xpt*-*pbuX* operon for xanthine salvage and catabolism, *guaA* encoding for GMP synthetase and the transporter gene *pbuG*. Putative PreQ1 riboswitches were identified upstream of the *queC*-*queD*-*queE* operon and the *queF* gene. Four putative c-di-GMP riboswitches precede genes of unknown function, the *sinI* gene encoding the antagonist of biofilm regulator SinR and the *BMMGA3*_*04760*-*BMMGA3*_*04765* operon which is composed of genes participating in c-di-GMP turn-over. The presence of a *glmS* riboswitch was detected in the 5′-UTR of *glmS* transcript.

**Table 4:** **Riboswitches identified in** ***B. methanolicus*** **MGA3 and their respective transcriptional organization, ligand and function**

**Riboswitch and its transcriptional organization**	**Ligand**	**Related function**	**Locus tag(s)**
(AdoCbl)^*^-*yvrC*-*yvrB*-*BMMGA3*_*01180*-*cobD*-*BMMGA3*_*01190*-*cobU*-*cobQ*	Adenosylcobalamin	Cobalamin biosynthesis and transport	*BMMGA3*_*01170- BMMGA3_01200*
(AdoCbl)-*metE*		B_12_-independent methionine synthase	*BMMGA3_08635*
(AdoCbl)-*nrdAB*		Ribonucleotide-diphosphate reductase	*BMMGA3_09290*
(FMN)-*ribD*-*ribE*-*ribBA*-*ribH*	Flavin mononucleotide	Riboflavin biosynthesis	*BMMGA3_09890-BMMGA3_09905*
(FMN)-*fmnP*		Riboflavin transport	*BMMGA3*_*10940*
(SAM)-*BMMGA3*_*009040*	S-adenosylmethionine	Unknown function	*BMMGA3*_*09040*
(SAM)-*metK*		S-adenosylmethionine synthesis	*BMMGA3*_*13565*
(SAM)-*cysH*-*sat*-*cysC*-*nirA*-*BMMGA3*_*02575*-*cobA*-*sirB1*-*ytnM*		Sulfur assimilation	*BMMGA3*_*02555*-*BMMGA3*_*02595*
(SAM)-*metN-metP-metQ*		Methionine ABC transporter	*BMMGA3_14365-BMMGA3_14375*
(SAM)-*mtnW*-*mtnX*-*mtnB*-*mtnD*		Methionine salvage	*BMMGA3*_*05045*-*BMMGA3*_*05060*
(SAM)-*BMMGA3*_*01840*-*BMMGA3*_*01845*-*BMMGA3*_*01850*		Unknown function	*BMMGA3*_*01840*-*BMMGA3*_*01850*
(SAM)-*dmoA*		DMS degradation	*BMMGA3*_*09055*
(SAM)-*mtnK*		Methionine salvage	*BMMGA3*_*05030*
(SAM)-*BMMGA3*_*08645*-*BMMGA3*_*08640*		Unknown function	*BMMGA3*_*08640*-*BMMGA3*_*08645*
(SAM)-*BMMGA3*_*03025*		Methionine biosynthesis	*BMMGA3*_*03025*
(SAM)-*BMMGA3*_*14380*		Methionine biosynthesis	*BMMGA3*_*14380*
(SAM)-*metI*-*metC*-*yitJ-metH*		Methionine biosynthesis	*BMMGA3*_*08600*-*BMMGA3*_*08615*
(SAM)-(PyrR)- *BMMGA3*_*09265*-*BMMGA3*_*09270*		Major Facilitator Superfamily (MFS) transporter	*BMMGA3*_*09265*-*BMMGA3*_*09270*
(SAM)-(PyrR)-*BMMGA3*_*03030*-*ssuA1*-*ssuC1*-*ssuB1*-*acsA1*-*thrA*		Sulfonate uptake	*BMMGA3*_*03030*-*BMMGA3*_*03055*
(SAM)-(PyrR)-*metN1*-*metP1*-*metQ1*		Methionine ABC transporter	*BMMGA3*_*08730*-*BMMGA3*_*08740*
(TPP)-*thiD*-*thiE*	Thiamine pyrophosphate	Biosynthesis of thiamine	*BMMGA3*_*03005*-*BMMGA3*_*03010*
(TPP)-*tenA*-*tauB*-*tauC*		ABC transporter	*BMMGA3*_*07350*-*BMMGA3*_*07360*
(TPP)-*thiW*		Thiamine uptake	BMMGA3_01780
(TPP)-*tauA*-*BMMGA3*_*09465*-*tauC*-*tauB*-*tatD*		ABC transporter	*BMMGA3*_*09450*-*BMMGA3*_*09470*
(TPP)-*tenI*-*thiO*-*thiS*-*thiG*-*moeB*		Biosynthesis of thiamine	*BMMGA3*_*01095*-*BMMGA3*_*01115*
(Gly)-*gcvT*-*gcvPA*-*gcvPB*	Glycine	Glycine utilization	*BMMGA3*_*11545*-*BMMGA3*_*11555*
(Gly)-*BMMGA3*_*03000*		Unknown function	*BMMGA3*_*03000*
(Lys)-*BMMGA3*_*01150*	Lysine	Arginine/ornithine antiporter	*BMMGA3*_*01150*
(Pur)-*pbuG*	Purines	Hypoxanthine and guanine uptake	*BMMGA3*_*01600*
(Pur)-*guaA*		Purine salvage	*BMMGA3*_*01595*
(Pur)-*purE*-*purK*-*purB*-*purC*-*purS*-*purQ*-*purL*-*purF*-*purM*-*purN*-*purH*		Purine biosynthesis	*BMMGA3*_*01635*-*BMMGA3*_*01690*
(Pur)-*xpt*-*pbuX*		Xanthine salvage and catabolism	*BMMGA3*_*10440*-*BMMGA3*_*10445*
(PreQ_1_)-*queF*	7-amminomethyl-7-deazaguanine	Queuosine biosynthesis	*BMMGA3_03100*
(PreQ_1_)-*queC*-*queD*-*queE*		Queuosine biosynthesis	*BMMGA3_09070-BMMGA3_09080*
(c-di-GMP)-*BMMGA3*_*15625*	Cyclic di-GMP	Unknown function	*BMMGA3*_*15625*
(c-di-GMP)-*BMMGA3*_*04760*-*BMMGA3*_*04765*		Intracellular signal transduction	*BMMGA3*_*04760*-*BMMGA3*_*04765*
(c-di-GMP)-*sinI*		Control of biofilm formation	*BMMGA3*_*08510*
(c-di-GMP)-*BMMGA3*_*15380*		Unknown function	*BMMGA3*_*15380*
(ydaO/yuaA)-*BMMGA3_02260*	Cyclic di-AMP	Membrane protein	*BMMGA3*_*02260*
(ydaO/yuaA)-*BMMGA3_14870*		Amino acid permease	*BMMGA3*_*14870*
(glmS)-*glmS*	Glucosamine-6-phosphate	Glutamine-fructose-6-phosphate transaminase	*BMMGA3*_*01020*

**Table 5:** **T-boxes identified in** ***B. methanolicus*** **MGA3 and their respective transcriptional organization, affected amino acid biosynthesis and related function**

**T-box and its transcriptional organization**	**Affected amino acid biosynthesis**	**Related function**	**Locus tag(s)**
(T-box)*-*alaS*	Alanine	aaRS	*BMMGA3_12250*
*gltX*-(T-box)-*cysE*-*cysS*-*mrnC*-*yacO*-*yacP*	Cysteine	Amino acid biosynthesis, aaRS, maturation of 23S rRNA	*BMMGA3_00580-BMMGA3_00605*
(T-box)-*glyQS*	Glycine	aaRS	*BMMGA3_11920*
(T-box)-*hisS*-*aspS*	Histidine and aspartate	aaRS	*BMMGA3_12305-BMMGA3_12310*
(T-box)-*ileS*	Isoleucine	aaRS	*BMMGA3_05760*
(T-box)-*leuS*	Leucine	aaRS	*BMMGA3_13510*
(T-box)-*pheS*	Phenylalanine	aaRS	*BMMGA3_12925*
(PyrR)(T-box)-*proI*	Proline	Amino acid biosynthesis	*BMMGA3_11220*
(PyrR)(T-box)-*serS*	Serine	aaRS	*BMMGA3_00090*
(T-box)-*thrS*	Threonine	aaRS	*BMMGA3_12975*
(T-box)(T-box)-*trpE*-*trpGD*-*trpD*-*trpF*-*trpB*-*trpA*	Tryptophan	Amino acid biosynthesis	*BMMGA3_09595-BMMGA3_09625*
(T-box)-*trpS*	Tryptophan	aaRS	*BMMGA3_04510*
(T-box)-*tyrS*	Tyrosine	aaRS	*BMMGA3_13290*
(T-box)-*valS*	Valine	aaRS	*BMMGA3_12655*
(T-box)-*brnQ*	Branched amino acids	Amino acid transport	*BMMGA3_02675*
(T-box)-*ilvB*	Branched amino acids	Amino acid biosynthesis	*BMMGA3_12765*
(T-box)-*cstA1*	-	Carbon starvation protein^§^	*BMMGA3_02765*

*(T-box) = T-box regulatory element; (PyrR) = PyrR binding site; aaRS = aminoacyl-tRNA synthetases; features of an operon are connected with a hyphen.

^§^The whole transcriptome data does not unambiguously clarify if the T-box belongs to the *cstA1* gene.

**Table 6:** **Ribosomal protein leaders identified in** ***B. methanolicus*** **MGA3 and their respective transcriptional organization and function**

**Ribosomal protein leaders and its transcriptional organization**	**Related function**	**Locus tag(s)**
(L10)*-*rplJ-rplL*	Ribosomal proteins	*BMMGA3_00640-BMMGA3_00645*
(L13)-*rplM-rpsI*	Ribosomal proteins	*BMMGA3_00865-BMMGA3_00870*
(L19)-*rplS*	Ribosomal protein	*BMMGA3_06025*
(L20)-*infC-rpmI-rplT*	Translation initiation factor IF-3, ribosomal proteins	*BMMGA3_12960-BMMGA3_12970*
(L21)-*rplU-BMMGA3*_*12500-rpmA*	Ribosomal proteins, protein of unknown function	*BMMGA3*_*12495*-*BMMGA3*_*12505*

*(L10) = L10 leader; (L13) = L13 leader; (L19) = L19 leader; (L20) = L20 leader; (L21) = L21 leader.

**Table 7:** **Other** ***cis-*** **regulatory RNA motifs identified in** ***B. methanolicus*** **MGA3 and their respective transcriptional organization and function**

***cis-*** **regulatory RNA motif and its transcriptional organization**	**Related function**	**Locus tag(s)**
(PyrR)*-*pyrR*-*pyrP*-*pyrB*-*pyrC*-*pyrAA*-*pyrAB*-*pyrK*-*pyrD*-*pyrF*-*pyrE*	Pyrimidine biosynthesis	*BMMGA3*_*05780*-*BMMGA3*_*05825*
(pan)-*panB*-*panC*-*panD*	Biosynthesis of coenzyme A	*BMMGA3*_*10610*- *BMMGA3*_*10620*
(yjdF)-*yjdF*	Unknown function	*BMMGA3*_*03360*
(yybP-*ykoY*)(PyrR)-*ykoY*	Membrane protein	*BMMGA3*_*14085*
(ylbH)^§^	Unknown function	-

*(PyrR) = PyrR binding site; (pan) = *pan* RNA motif; (yjdF) = *yjdF* RNA motif; yybP-ykoY = *yybP*-*ykoY* RNA motif; (ylbH) = *ylbH* RNA motif.

^§^A CDS for a conserved hypothetical protein is located on the opposite strand between the ylbH RNA motif and the next downstream gene.

Another major group of *cis-*regulatory elements in *B. methanolicus* MGA3 are T-boxes [46] that amount for 26.6% of all detected *cis-*regulatory RNA motifs (Table 5). Eighteen T-boxes were identified upstream of genes or operons encoding aminoacyl-tRNA synthetases, proteins involved in amino acid biosynthesis and transport (Table 5). Five auto-regulatory leader structures of ribosomal protein genes [47] were found (Table 6). Eleven further *cis-*regulatory RNA motifs were uncovered including a PyrR binding site preceding the *pyr* operon responsible for pyrimidine biosynthesis, *pan*, *yjdF*, and *yybP*-*ykoY* RNA motifs upstream of the *panB* operon, *yjdF* and *ykoY*, respectively, as well as the *ylbH* leader (Table 7).

### Transcript abundances of *B. methanolicus* MGA3

All 3,225 loci for annotated chromosomal genes present in the genome were analyzed with respect to their transcript abundances in the cells. Normalization by calculating log-RPKM values allowed comparison between genes as well as within one or different datasets (see section “Calculation of transcript abundances in *Bacillus methanolicus* MGA3”). Transcripts were detected for 2,529 of 3,225 gene loci (78.4%). Genes that do not have an annotated function or encode hypothetical proteins are overrepresented among the non-transcribed genes (64.2% of the non-transcribed as compared to 23.4% of all genes). Similarly, short ORFs up to 300 nt are over-represented among the non-transcribed genes (34.7% as compared to 11.1%). Abundances of the transcribed genes were arbitrarily divided into 4 classes: ‘Low’, ‘Middle’, ‘High’ and ‘Very high’. The genes with ‘Very high’ transcript abundance constitute 1.3% of all chromosomal genes (Table 8). Of those 41 genes, 17 genes encode proteins related to transcription, translation or chaperone-assisted degradation, which is typical in bacteria [48]. Four genes with ‘Very high’ transcript abundance encode the methylotrophic enzymes 3-hexulose-6-phosphate synthase (*hps*), 6-phospho-3-hexuloisomerase (*phi*), 6-phosphofructokinase (*pfk*) and ribose 5-phosphate isomerase (*rpi*). All those genes encode proteins of the RuMP and the high abundance of their transcripts may be due to the fact that methanol was used in most cultivations from which RNA was initially prepared for RNA-seq analysis.

**Table 8:** **Highly abundant transcripts of** ***B. methanolicus*** **MGA3** ^**a**^

**Gene name** ^**b**^	**Gene product**	**log-RPKM**	**Functional category**
*hps*	3-hexulose-6-phosphate synthase	12,308	Carbohydrate transport and metabolism
*phi*	3-hexulose-6-phosphate isomerase	8,086	Carbohydrate transport and metabolism
*rpiB*	Ribose 5-phosphate isomerase	3,473	Carbohydrate transport and metabolism
*pfkC*	6-phosphofructokinase	1,695	Carbohydrate transport and metabolism
*BMMGA3_16000*	F0F1 ATP synthase subunit B	1,840	Energy production and conversion
*yumB*	NADH dehydrogenase-like protein YumB	1,689	Energy production and conversion
*gltB*	Glutamate synthase [NADPH] small chain	3,302	Amino acid transport and metabolism
*ilvE*	Putative aminotransferase	3,134	Amino acid transport and metabolism
*argG*	Argininosuccinate synthase	2,738	Amino acid transport and metabolism
*argF*	Ornithine carbamoyltransferase	2,434	Amino acid transport and metabolism
*ilvC*	Ketol-acid reductoisomerase	2,265	Amino acid transport and metabolism
*argB*	Acetylglutamate kinase	2,207	Amino acid transport and metabolism
*argH*	Argininosuccinate lyase	2,187	Amino acid transport and metabolism
*BMMGA3_12455*	L-aspartate oxidase	2,028	Amino acid transport and metabolism
*gltA*	Glutamate synthase [NADPH] large chain	1,780	Amino acid transport and metabolism
*cysK*	Cysteine synthase	1,758	Amino acid transport and metabolism
*BMMGA3_01295*	Putative transcriptional regulator, CopG family	2,378	Transcription
*rpoA*	DNA-directed RNA polymerase subunit alpha	1,670	Transcription
*rpmC*	50S ribosomal protein L29	7,670	Translation
*BMMGA3_00355*	hypothetical protein	4,650	Translation
*rplR*	50S ribosomal protein L18	4,412	Translation
*rpsG*	30S ribosomal protein S7	3,468	Translation
*rpl14e*	50S ribosomal protein L14e	2,607	Translation
*rpsJ*	30S ribosomal protein S10	2,511	Translation
*rplS*	50S ribosomal protein L19	2,249	Translation
*rpsS*	30S ribosomal protein S19	2,204	Translation
*rplW*	50S ribosomal protein L23	2,004	Translation
*ybxF*	50S ribosomal protein L7Ae	1,977	Translation
*rpsU*	30S ribosomal protein S21	1,786	Translation
*BMMGA3_12500*	hypothetical protein	1,733	Translation
*rplV*	50S ribosomal protein L22	1,710	Translation
*ykuU*	Thioredoxin-like protein	2,670	Posttranslational modification, secretion, and vesicular transport
*groES*	co-chaperonin GroES	2,498	Posttranslational modification, secretion, and vesicular transport
*sodA*	Superoxide dismutase [Mn]	2,511	Inorganic ion transport and metabolism
*cysC*	Adenylyl-sulfate kinase	1,925	Defense mechanisms
*BMMGA3_06005*	hypothetical protein	5,873	Function unknown
*ssb*	Single-stranded DNA-binding protein	4,261	Function unknown
*BMMGA3_02575*	hypothetical protein (duf3906)	1,757	Function unknown
*BMMGA3_03940*	small, acid-soluble spore proteins	1,728	Function unknown
*rny*	Ribonuclease Y	2,180	General function prediction only
*BMMGA3_15110*	acetyltransferase	2,110	General function prediction only

^a^Transcript abundance was arbitrarily classified into 5 classes. The RNA abundance is reflected by the log-RPKM value. The data set revealed 21.6% non-transcribed genes, 3.8% genes with low RNA abundance (log-RPKM >0-16), 41.2% genes with middle (log-RPKM >16-160), 32.2% genes with high (log-RPKM >160-1600) and 1.3% genes with very high (log-RPKM >1600) transcript abundance.

^b^The genes are sorted according to their functional category.

## Discussion

In the present study, the complete transcriptome of *B. methanolicus* MGA3 was analyzed for the first time with RNA sequencing. Previously, its genome including the plasmids pBM19 and pBM69 was sequenced [11,16] and approximately 1000 proteins were characterized by label-free quantitative proteomics [8]. Here, a comprehensive characterization of transcription start sites, conserved sequence motifs of promoter and translation initiation sequences, the transcriptional organization of genes and putative regulatory RNA elements of *B. methanolicus* MGA3 using two different RNA-seq approaches is presented.

### Analysis of 5′-UTRs in *B. methanolicus* MGA3

The distances of 1,642 TSSs to the next TLSs were equal or longer than 10 nucleotides in > 99% cases. The distance distribution showed a maximum at 26–35 nucleotides (22%) before decreasing exponentially with increasing 5′-UTR lengths. This distance distribution is typical for bacteria that feature 5′-UTRs shorter than 100 bp and peak at a length of around 25–35 nt [17,49,50]. Similarly, the low number of leaderless transcripts in *B. methanolicus* MGA3 (6 with a 5′-UTR ≤ 10) is in accordance with *in silico* predictions for 953 bacterial genomes that revealed only about 25% of species belonging to the phylum *Firmicutes* possess leaderless transcripts and in these species 2.4% to 16.6% of all sequences are leaderless [51]. Experimental evidence supports this notion (*e.g.* 2.1% leaderless transcripts in *Bacillus licheniformis* [52]). Contrary, leaderless transcripts are abundant in species of the phyla *Actinobacteria* and *Deinococcus*-*Thermus*, *e.g. Actinoplanes* sp. SE50/110 (20%) [49]*.* The exact translation mechanism of leaderless transcripts without a complete RBSs is still not fully elucidated, however, in those transcripts close proximity of the start codon to the 5' terminus is important for both ribosome binding and translation [53].

### Characteristics of translation start sites, ribosome binding sites, promoters and operons in *B. methanolicus* MGA3

The most frequent initiation codon in *B. methanolicus* MGA3 is ATG (75.5%), a frequency typical for 620 complete bacterial genomes (80.1%; [54]). As in *B. subtilis* [55], the generally uncommon initiation codon TTG (13.9%) was more common than GTG (10.6%) in *B. methanolicus* MGA3.

Besides the initiation codon, the efficiency of translation depends on the interaction between the RBS and the 3′-end of the 16S rRNA [56]. The conserved RBS motif aGGaGg in *B. methanolicus* MGA3 is reverse complementary to the 3′-end of the 16S rRNA. The determined sequence motif is identical with the bacterial consensus sequence AGGAGG [57] and was found in 21.9% of the upstream regions. The first three bases of the motif are conserved in 66.9% of the upstream sequences, which is in line with the notion that the first three bases of the RBS are potentially more relevant to the translation initiation *via* hybridization to the 3′-terminus of the 16S rRNA, while the other bases possibly only modulate the translation efficiency [58]. The spacing between translation starts and ribosome binding sites is known to affect translation efficiency [58]. In *B. methanolicus* MGA3, the distance between both elements was between 5 to 10 nucleotides (97.1%) with an average of 7.4 bases, thus, matching the optimal spacing determined for *E. coli*, *B. subtilis* and various other bacteria [59,60].

Conserved promoter motifs were determined upstream of the exact TSSs positions within the *B. methanolicus* MGA3 and revealed a -10 region (TAtaaT), which is identical to the −10 consensus sequence TATAAT originally described for *E. coli σ*^70^-RNAP [61] and is also recognized by the *B. subtilis σ*^A^-RNAP [62]. The finding that TGN is preceding 33% of the −10 motifs in *B. methanolicus* MGA3 is in accordance with extended −10 motifs of *σ*^70^/*σ*^A^-dependent promoters in *E. coli* and *B. subtilis* with the dinucleotide TG located 1 bp upstream of the core hexamer [63,64]. Extended -10 promoters specifically interact with the conserved sigma factor region 3.0 to form the polymerase-promoter complex and often lack the -35 consensus element [65]. While it is assumed that the additional contact with the sigma factor in extended -10 promoters compensates for the interaction between the region 4.2 and the −35 element [66], the -35 sequence in *B. methanolicus* was not particularly less conserved if a TGN extended −10 region was present. The so-called discriminator element downstream of the -10 hexamer may play a role in the regulation of the stringent response during transition from exponential to stationary phase [67] and nucleotides at positions -6 to -4 interact with the sigma factor region 1.2 [68]. However, in *B. methanolicus* MGA3 only two consecutive adenines are weakly conserved, which reflects the overall low G + C content of this bacterium (38.6%). The mean spacing of the identified -10 regions to the TSS is with 6.7 bases in typical range for bacteria [30,63]. The average spacing of 16.6 nt between the −10 and the −35 regions is very close to 17, the optimal spacing determined for these elements in *E. coli* and *B. subtilis* [63,69]. The weakly conserved −35 hexamer ttgana determined for *B. methanolicus* MGA3 resembles the −35 consensus sequence TTGACA present in *E. coli*, *B. subtilis* and numerous other species [69,70].

Although various different stress conditions were included in the experimental setup, the analysis of conserved promoter motifs in *B. methanolicus* MGA3 only returned sequences that are identical to the consensus sequences recognized by bacterial housekeeping sigma factors. This is likely due to the fact that in all tested conditions the number of housekeeping genes accounts for most of the identified TSSs and thus overlays the more rare upstream regions of stress-induced transcripts. Since pooled RNAs were sequenced, it was not possible to differentiate between transcripts which were resulting from a certain cultivation condition. To identify sigma factor-dependent promoter motifs, RNA-seq data sets for individual growth or stress conditions need to be generated and analyzed; however, this was not within the scope of the present study.

In *B. methanolicus* MGA3, 381 primary operons (encompassing 1,164 genes) with 94 internal suboperons were identified, while 940 genes were transcribed monocistronically under the chosen growth/stress conditions. Although this distribution is typical and also found in *E. coli*, *B. subtilis* and *C. glutamicum* [17,71,72], it is important to mention here that the transcriptional organization of genes is very likely not a static feature necessitating the analysis of RNA-seq data sets for individual growth or stress conditions.

### Description of novel transcripts in *B. methanolicus* MGA3

The novel transcripts detected in *B. methanolicus* MGA3 were classified as intergenic, intragenic or antisense transcripts (Table 3 and Additional file 4: Table S4; Additional file 5: Table S5 and Additional file 6: Table S6). The 27 novel intergenic transcripts represent small RNA and small protein genes, but their function still has to be elucidated. For six small RNAs (sRNA) and one small protein of *B. methanolicus* MGA3 a function could be predicted *via* database searches. The scRNA (337 nucleotides in *B. methanolicus* MGA3) is a member of the signal-recognition-particle-like RNA family [43]. Together with the Ffh protein component (encoded in *B. methanolicus* MGA3 by *BMMGA3_05995*), the scRNA constitutes the bacterial signal recognition particle (SRP), which targets proteins for secretion by directing them to the translocation channels in the membrane [73]. The RNase P is an essential and ubiquitous ribozyme, mainly responsible the maturation of tRNA molecules [74]. Bacterial RNase P consists of two components: RNase P RNA, which is the catalytic subunit, and the C5 protein which assists in the release of the product from the holoenzyme [75] and is encoded in *B. methanolicus* MGA3 by *BMMGA3_16755*. The 6S RNA, which typically regulates transcription by forming a stable complex with the housekeeping form of the RNAP holoenzyme [76], is present in two copies in the *B. methanolicus* MGA3 genome, which is consistent with findings from *B. subtilis* and several closely related bacteria [77]. Intriguingly, these two copies are differentially expressed in these organisms, which suggests their involvement at different stages of growth [77]. *B. methanolicus* MGA3 possesses transfer-messenger RNA (tmRNA) as one of the most abundant RNA species, similarly to other bacterial species [78]. The tmRNA can act as a tRNA and mRNA and constitutes a ribonucleoprotein complex together with the three protein components SmpB, EF-Tu and (usually) the ribosomal protein S1, while the latter is absent from bacilli and is substituted by a homologue encoded by the *ypfD* gene [79]. The bacterial ribonucleoprotein complex resets stalled ribosomes and a tmRNA-encoded signal peptide is cotranslationally appended to the C-termini of nascent polypeptide and serves as a recognition signal for degradation [80]. The tmRNA open reading frame in *B. methanolicus* MGA3 encodes a 16 amino acid (aa) signal peptide with a terminal stop codon (KTSKPITGNQKLALAA-Stop), which resembles the 15 aa sequence identified in *B. subtilis* (AGKTNSFNQNVALAA-Stop) [81]. *B. methanolicus* MGA3 may possess a type I toxin-antitoxin system with toxin-encoding gene *bsrG* and the SR4 RNA as initially described in *B. subtilis* [44]. In *B. subtilis,* the small RNA SR4 promotes the degradation of the *bsrG* mRNA *via* the double-strand specific RNase III, while the absence of SR4 lead to the overexpression of *bsrG* and subsequently to cell lysis and growth retardation [44]. A comparable function may be predicted for *B. methanolicus* MGA3 since *bsrG* and SR4 RNA are located antisense to each other with an overlap of ca. 120 bases and the 43 aa toxin encoded by *bsrG* in *B. methanolicus* MGA3 (MTAVLQHRRSLAIVVPAGVRPMKQDRPLPQFAVKGGLFILVKS-Stop) shares 19% of identity and 31% of similarity with the 38 aa toxin (MTVYESLMIMINFGGLILNTVLLIFNIMMIVTSSQKKK-Stop) present in *B. subtilis*.

The frequent occurrence of intragenic TSSs (10%) in *B. methanolicus* MGA3 that almost exclusively (99%) belong to intragenic sense transcripts preceded by conserved −35 and −10 motifs for the housekeeping sigma factor is not unusual in bacteria. Notably, about one third of the internal sense transcripts in *B. methanolicus* MGA3 belong to suboperons, thus, the remaining downstream coding sequences are transcribed completely. The functional role of the remaining intragenic transcripts, for example as alternative, shorter proteins, novel or processed RNAs which increase the transcriptomic complexity, is not clear and should be further analyzed.

Altogether, 152 antisense transcripts longer than 20 nt were identified in 146 different protein-coding genes of *B. methanolicus* MGA3. Extrapolated, 4.4% of the *B. methanolicus* MGA3 genes or more may be subject to antisense transcription. This number is in accordance with findings for other species from the genus *Bacillus* [52] and other transcriptome studies which suggest that antisense transcription probably affects 5-20% of bacterial genes and in some cases even considerably more, *i.e.* about 46% in *Helicobacter pylori* [82]. A conserved housekeeping −10 motif was identified for the antisense transcripts in *B. methanolicus* MGA3 in their upstream regions. By contrast, neither a conserved -35 motif nor an extended −10 motifs were found for these antisense transcripts. Antisense transcripts may regulate gene expression either by hybridization with part or the complete target sequence and *e.g.* blocking/releasing ribonuclease cleavage or ribosome binding sites [83] or competing with transcription of sense and antisense sequences [84]. It is predicted that transcription of 11 antisense genes of *B. methanolicus* MGA3 interferes with transcription of the neighboring gene due to overlapping 5′-UTRs. Antisense transcription has been detected in all domains of life and therefore likely represents a common form of gene expression regulation [83,85]. Although, RNA-seq studies often reveal large numbers of antisense transcripts, further validation and functional characterization is necessary to identify if, and to what extent, those antisense RNAs participate in gene regulation.

### Discovery of *cis*-regulatory RNA elements in the *B. methanolicus* MGA3 genome

*Cis-*regulatory RNA elements belong to broad class of non-coding RNA motifs which are present upstream of the regulated genes. In the present study, 75 *cis-*regulatory elements were detected in 68 different 5′-UTRs in the transcriptome of *B. methanolicus* MGA3. The RNA-seq data of *B. methanolicus* reveal three cobalamin riboswitches upstream of genes/operons predicted to encode cobalamin biosynthesis- and transport-related proteins, B_12_-independent methionine synthase and ribonucleotide-diphosphate reductase. Likely, they function as demonstrated for the cobalamin riboswitches upstream of the *nrdABS* operon of *Streptomyces coelicolor* and of the B_12_-independent methionine synthase of *Mycobacterium tuberculosis* [86,87]. The organization of FMN, SAM, TPP, lysine and purine riboswitches found in the genome of *B. methanolicus* MGA3 is similar to those of other bacteria [88-91]. The function of the riboswitch belonging to the *gcvT* operon in the genome of *B. methanolicus* has been confirmed experimentally for *B. subtilis.* A next ligand for the riboswitch found in the genome of *B. methanolicus* is PreQ_1_ (7-aminomethyl-7-deazaguanosine) which is a precursor of nucleoside Q (queuosine). This modified guanine nucleotide present in some tRNA molecules is known to participate in the regulation of genes involved in queuosine biosynthesis by interaction with PreQ_1_ riboswitches in other bacterial species [92,93]. The intracellular signal molecule bis-(3′-5')-cyclic dimeric guanosine monophosphate (c-di-GMP) is found in many bacteria and may be involved *e.g.* in regulation of motility, virulence and biofilm formation (as reviewed in [94]) and c-di-GMP riboswitches have been characterized [95]. Besides presence of putative c-di-GMP riboswitches the genome of *B. methanolicus* encodes at least 8 enzymes for synthesis and degradation of c-di-GMP (*BMMGA3_05195*, *BMMGA3_05210*, *BMMGA3_09025*, *BMMGA3_10495*, *BMMGA3_12645*, *BMMGA3_13275*, *BMMGA3_14250* and *BMMGA3_15230*). The *ydaO/yuaA* leader was shown to interact with cyclic di-AMP in *B. subtilis* [96]. This motif is present upstream of two genes of *B. methanolicus* MGA3, *BMMGA3_02260* and *BMMGA3_14870,* which encode a membrane protein and an amino acid permease, respectively. Unlike the other riboswitches mentioned above, the *glmS* ribozyme, which is present in *B. methanolicus* MGA3, is both a ribozyme, catalyzing a chemical reaction, and a riboswitch, regulating transcription of the glutamine-fructose-6-phosphate transaminase gene as a function of the glucosamine-6-phosphate (GlcN6P) concentration [97]. This type of regulation involves self-cleavage of the *glmS* ribozyme in the presence of GlcN6P. Although homologs are found in at least 17 further Gram-positive bacteria the function of the predicted *glmS* riboswitch in *B. methanolicus* has yet to be proven [97].

In addition to the riboswitches, one of the most abundant regulatory elements discovered for *B. methanolicus* are T-box motifs which likely control gene expression by interacting with uncharged tRNA molecules. The binding of uncharged tRNA prevents formation of a translation terminator and, thus enables translation [98]. T-boxes were found in several monocistronic and polycistronic transcripts which feature genes encoding aminoacyl-tRNA synthetases (aaRS) or proteins involved in amino acid biosynthesis and transport in *B. methanolicus* MGA3. A tandem T-box was detected for the *trp*E operon which is also characteristic for certain *Bacillales* and some related species such as *Clostridium beijerinckii* and *Desulfitobacterium hafniense* [98]*.* The operon *hisS*-*aspS* represents an unusual case of two *aaRS* genes controlled by only one riboswitch. It has been suggested that this regulatory element can adopt two alternative secondary structures and therefore sense both tRNA_His_ and tRNA_Asp_ molecules [99]. Interestingly, the T-box was also found upstream of the gene coding for carbon starvation protein CstA1. However, the detailed investigation of whole transcriptome data for upstream region of the *cstA1* gene indicates that the T-box in fact might not regulate the CstA1 expression and is only a residue of a different transcription unit which was split in the process of genome rearrangements. This hypothesis is based on the fact that there is a break in the transcription between the TSS and the gene start codon in the whole transcriptome track. However, this assumption has yet to be confirmed experimentally.

Besides low-molecular-weight compounds, also proteins such as the ribosomal proteins L10, L13, L20, L21 and L29 are able to bind to the leader sequences of their own transcripts causing premature transcription termination at the leader terminator [100-102]. In *B. methanolicus* MGA3, five leader motifs characteristic for the genes of above-mentioned ribosomal proteins were detected. Additionally, a PyrR binding site was found in the leader sequence of the *pyr* operon. PyrR is encoded by and auto-regulates the pyrimidine biosynthesis operon by formation of a terminator and decrease of operon expression in *B. subtilis* [33].

In seven cases, *B. methanolicus* features two *cis-*regulatory RNA elements in the same upstream region: a duplicated T-box and in six cases the PyrR binding site was accompanied by either a SAM riboswitch (three times), a T-box (twice) or the *yybP*-*ykoY* leader (once). The presence of two consecutive *cis-*regulatory elements was observed in different bacterial species where it was proposed that the tandem arrangement leads to an increase of the regulatory capacity [103,104]. Interestingly, no known regulatory RNA element was found in the second longest leader sequence detected in the transcriptome of *B. methanolicus* MGA3. This 5′-UTR of 878 nt length belongs to the gene encoding molybdopterin dehydrogenase FAD-binding protein. A nucleotide BLAST analysis of the leader sequence showed only partial similarity to the upstream region of molybdopterin dehydrogenase FAD-binding protein gene in the *Geobacillus stearothermophilus* NUB3621 genome. However, because of the low conservation of the motif among bacterial species no conclusions about the function of the long leader can be drawn and further experiments need to be performed to understand its role in the transcriptome of *B. methanolicus* MGA3.

### The mRNA abundances in *B. methanolicus* MGA3 reflect its metabolic organization

Under the chosen conditions, 21.6% of the chromosomal genes of *B. methanolicus* MGA3 were not transcribed. The RNA-seq data were generated from a pool of RNA preparations from various conditions, however, the different lab conditions most likely reflect only a small subset of the conditions encountered by *B. methanolicus* in its natural habitat. Alternatively, since genes for hypothetical proteins are about three times more abundant in the group of non-transcribed genes (64.2%) than in the group of genes for which transcripts were detected (23.4%), false annotations of protein-coding genes may have increased the fraction of non-transcribed genes.

As a first approximation to categorize expression levels, all chromosomal genes of *B. methanolicus* MGA3 were divided into four groups according to arbitrarily chosen transcripts abundance thresholds (section “Transcript abundances of *B. methanolicus* MGA3”). The 41 genes showing the highest RNA abundance encoded proteins of (1) amino acid metabolism and transport, (2) transcription, translation and post-translational modification, (3) carbohydrates metabolism and energy production, and (4) other functions. This representation of the gene functions is expected and to some extend corresponds to the results obtained on proteome level for *B. subtilis* [105]. Notably, genes relevant for methylotrophy of *B. methanolicus* MGA3 stood out. Although, the results obtained in the present study do not allow to perform the direct comparison of transcripts abundance between different references (chromosome, plasmids pBM19 and pBM69) [106], the pBM19-encoded *mdh* has the most abundant transcript among all pBM19-encoded genes, while the chromosome-encoded methanol dehydrogenase genes have rather low transcript abundances. This finding is in accordance with qRT-PCR experiments and the proteome analyses that showed that the pBM19-encoded *mdh* transcript and gene product is more abundant than those of the chromosomal genes *mdh2* (60 -fold) and *mdh3* (4000-fold) [106]. Formaldehyde, a toxic intermediate generated by methanol oxidation, is assimilated via 3-hexulose-6-phosphate synthase and 6-phospho-3-hexuloisomerase. As expected, the co-transcribed *hps* and *phi* genes showed the highest RNA abundance of all the chromosomal genes. For 6-phospofructokinase which catalyzes the next step in the RuMP cycle, high transcript abundance for the chromosomal gene was observed. Transcripts of the chromosomal genes encoding fructose 1,6-bisphosphate aldolase [14], transketolase [12], fructose 1,6-bisphosphatase [13] and ribose 5-phosphate epimerase belong to the transcript group with high abundance. The last reaction of the RuMP cycle is catalyzed by ribose-5-phosphate isomerase, which is encoded only on the chromosome. As for *hps* and *phi* that are also only encoded on the chromosome, *rpi* RNA was very abundant. Interestingly, the *gltAB* operon encoding glutamate synthase was also found among the highly abundant genes (*gltA* and *gltB*); *B. methanolicus* MGA3 has two alternative active glutamate synthases [106] and this finding may suggest that the *gltAB* encoded enzyme is important for the high production of L-glutamate by this bacterium. In future work aimed to gain an in-depth understanding of methylotrophy in *B. methanolicus*, it will be imperative to combine transcriptome, proteome and metabolome experiments under carefully controlled conditions of growth with methanol *versus* a non-methylotrophic carbon source.

## Conclusion

In this study, RNA-seq data sets have been generated for the primary and the whole transcriptome of the thermophilic methylotrophic *Bacillus methanolicus* MGA3. Altogether, 1,642 TSSs in the upstream regions of annotated genes were identified and used for analysis of the 5′-UTR length distribution and the detection of 75 *cis-*regulatory RNA elements. Additionally, the exact TSSs were used to detect conserved sequence motifs for TLSs, RBSs and promoters. Examination of the whole transcriptome enabled the validation of 365 novel transcripts, the uncovering of 381 operons and the determination of the mRNA abundance in *B. methanolicus* MGA3. The data established in this study deliver valuable insight regarding various transcriptomic elements and represent the basis for further transcriptome studies of *B. methanolicus* MGA3. Moreover, detailed insight into promoter and translation initiation sequences should be valuable for the future development of new and better expression tools for *B. methanolicus*.

## Availability of supporting data

The data sets supporting the results of this article are available in the NCBI Gene Expression Omnibus database, under the accession number GSE64469, http://www.ncbi.nlm.nih.gov/geo/query/acc.cgi?acc=GSE64469.

## Additional files

Additional file 1: Table S1. Primers used for RNA quality control. Additional file 2: Table S2.
*Bacillus methanolicus* MGA3 cultivation parameters. Additional file 3: Table S3. List of *Bacillus methanolicus* MGA3 CDS with corrected translational start sites. Additional file 4: Table S4. Novel transcripts identified in *Bacillus methanolicus* MGA3 with unknown function. Additional file 5: Table S5. Identified intragenic sense transcripts of *Bacillus methanolicus* MGA3. Additional file 6: Table S6. Identified antisense transcripts of *Bacillus methanolicus* MGA3.
